# Supplementary material for: Differential Diagnosis of Hyperferritinemia in Critically Ill Patients
Source: J Clin Med. 2022 Dec 27;12(1):192. doi: 10.3390/jcm12010192 (PMC9821140; doi:10.3390/jcm12010192)
Supplement: Supplementary file 1 [file jcm-12-00192-s001.zip › jcm-2082344-supplementary.pdf]

Supplement

Supplement Table S1. ICD-10 codes used for classification of different disease groups with corresponding numbers of patients.

**Liver disease**	**K76.1 (30)**	**Q44.6 (8)**	**Z75.77 (24)**	**K70.0 (13)**	**K70.2 (1)**	**K70.3 (103)**	**K70.4 (19)**	**K70.9 (1)**
**(760)**		**K71.0 (22)**	**K71.1 (5)**	**K71.7 (10)**	**K71.88 (4)**	**K71.9 (9)**	**K72.0 (432)**	**K72.1 (22)**
		**K72.71 (43)**	**K72.72 (43)**	**K72.73 (56)**	**K72.74 (80)**			
	**K72.79 (146)**	**K72.9 (39)**	**K74.0 (7)**	**K74.3 (6)**	**K74.4 (5)**	**K74.5 (2)**	**K74.6 (134)**	**K75.8 (9)**
		**K76.0 (91)**	**K76.5 (12)**	**K76.7 (84)**	**K76.8 (74)**	**K76.9 (10)**	**K77.8 (9)**	**O14.2 (3)**
		**R17 (12)**						
**Renal disease**	**I12.00 (21)**	**I12.01 (21)**	**I13.00 (4)**	**I13.01 (1)**	**I13.10 (3)**	**I13.11 (4)**	**I13.20 (25)**	**I13.21**
**(1820)**	**(25)**	**I15.11 (19)**	**N00.8 (3)**	**N00.9 (1)**	**N01.7 (7)**	**N01.8 (2)**	**N01.9 (3)**	**N03.5 (2)**
		**N03.8 (2)**	**N03.9 (4)**	**N04.3 (1)**	**N05.0 (2)**			
	**N05.4 (1)**	**N05.5 (1)**	**N05.8 (4)**	**N08.0 (9)**	**N08.1 (2)**	**N08.2 (9)**	**N08.3 (111)**	**N08.4 (6)**
		**N08.5 (43)**	**N10 (15)**	**N11.1 (3)**	**N13.3 (62)**	**N13.9 (19)**	**N17.0 (15)**	**N17.03 (12) N17.1**
	**(3)**	**N17.8 (396)**	**N17.81 (17)**	**N17.82 (42)**				

	N17.83 (307)	N17.89 (33)	N17.9 (415)	N17.91 (51)	N17.92 (43)	N17.99 (23)	N18.0 (160)	N18.1 (9)
		N18.2 (51)	N18.3 (218)	N18.4 (105)	N18.5 (297)	N18.82 (27)	N18.83 (63)	N18.84 (27)
		N18.89 (17)	N18.9 (27)	N19 (41)	N25.8 (146)			
	N25.9 (1)	N26 (20)	N28.8 (19)	N28.9 (3)	N99.0 (75)	O26.81 (3)	O90.4 (4)	Q61.2 (9)
		Q61.3 (3)	Q61.5 (1)	Q61.8 (2)	Z49.0 (26)	Z49.1 (15)	Z90.5 (38)	Z99.2 (37)
Autoimmune	M06.99 (20)	M30.0 (2)	E06.3 (12)	G35.10 (3)	I73.0 (11)	K50.0 (12)	K50.82 (1)	K50.88 (7)
disease (185)		K50.9 (7)	K51.9 (2)	L10.0 (2)	L12.0 (4)	L12.8 (1)	L13.9 (1)	L20.8 (7)
		L40.8 (12)	M05.30 (1)	M05.90 (1)	M06.10 (5)			
	M06.80 (4)	M06.90 (3)	M08.29 (1)	M13.0 (3)	M31.3 (27)	M31.7 (3)	M32.0 (2)	M32.1 (19)
		M32.8 (8)	M32.9 (3)	M33.9 (2)	M34.0 (1)	M34.1 (1)	M34.8 (4)	M34.9 (1)
		M35.0 (9)	M35.3 (3)	M35.8 (2)				
	M45.00 (3)	M45.09 (6)	M79.09 (1)					
Hepatitis (159)	B15.9 (6)	B16.2 (5)	B16.9 (9)	B17.2 (6)	B18.1 (27)	B18.2 (53)	K70.1 (5)	K71.6 (9)
		K73.1 (1)	K73.2 (2)	K73.8 (1)	K75.2 (2)	K75.4 (11)	Z22.5 (46)	
Tuberculosis (20)	A15.0 (4)	A15.1 (5)	A15.4 (1)	A15.6 (2)	A16.2 (7)	A16.9 (4)	A18.8 (1)	M90.08 (1)

HIV (70)	B20 (46)	B21 (4)	B22 (8)	B23.0 (2)	B23.8 (31)	B24 (15)	R75 (3)	U60.1 (8)
		U60.3 (49)	U60.9 (6)	U61.1 (8)	U61.2 (8)	U61.3 (47)	U61.9 (5)	Z21 (13)
HSV (107)	A60.0 (7)	A60.9 (2)	B00.1 (32)	B00.4 (6)	B00.7 (27)	B00.8 (42)		
CMV (139)	B25.0 (38)	B25.8 (8)	B25.80 (14)	B25.88 (61)	B25.9 (34)			
EBV (40)	B27.0 (39)	D82.3 (1)						
VZV (31)	B01.1 (1)	B02.2 (3)	B02.3 (2)	B02.8 (9)	B02.9 (20)			
Influenza (58)	J09 (35)	J10.0 (21)	J10.1 (3)	J10.8 (1)	U69.20 (28)			
Malaria (4)	B50.8 (4)	B54 (1)						
Bacterial,	J15.1 (283)	J15.2 (254)	J18.8 (242)	J18.9 (279)	J69.0 (179)	N39.0 (669)	U69.00 (1242)	I51.4 (6)
viral or fungal		J04.1 (11)	K61.1 (1)	A02.0 (6)	A04.7 (137)	A04.70 (43)	A04.79 (11)	A04.8 (14)
infection (2250)		A06.9 (1)	A08.0 (4)	A08.1 (10)	A08.3 (1)			
	A08.4 (1)	A09 (12)	A09.0 (24)	A09.9 (85)	A37.0 (1)	A42.0 (1)	A46 (45)	A48.1 (14)
		A49.0 (42)	A49.1(5)	A49.8 (40)	A49.9 (5)	A50.0 (3)	A52.1 (2)	A53.9 (2)
		A69.2 (1)	B37.0 (104)	B37.1 (260)	B37.2 (22)			

B37.3 (21)	B37.4 (95)	B37.6 (3)	B37.81 (48)	B37.88 (231)	B37.9 (26)	B39.4 (2)	B44.1 (105)
	B44.8 (17)	B44.9 (9)	B45.1 (1)	B46.5 (2)	B48.8 (20)	B49 (65)	B58.2 (7)
	B58.8 (1)	B58.9 (3)	B85.0 (5)	B95.7 (197)			
B97.8 (7)	G00.3 (6)	G00.8 (8)	G04.2 (7)	G04.9 (10)	G05.2 (10)	G06.0 (13)	G06.1 (10)
	H10.0 (3)	H10.8 (9)	H10.9 (2)	H20.0 (1)	H32.0 (3)	H44.1 (4)	H65.1 (3)
	H66.4 (1)	H66.9 (3)	H70.0 (14)	I30.1 (7)			
I30.9 (5)	I33.0 (86)	I38 (12)	I39.1 (18)	I40.0 (2)	I41.8 (3)	I43.0 (15)	J01.0 (11)
	J01.1 (1)	J01.2 (7)	J01.3 (15)	J02.8 (2)	J02.9 (4)	J03.8 (2)	J06.0 (3)
	J11.0 (3)	J12.8 (5)	J13 (56)	J14 (19)			
J15.0 (174)	J15.3 (4)	J15.4 (24)	J15.5 (122)	J15.6 (236)	J15.7 (5)	J15.8 (139)	J16.0 (9)
	J16.8 (63)	J17.0 (14)	J17.2 (343)	J17.3 (41)	J17.8 (28)	J18.0 (58)	J18.1 (112)
	J18.2 (74)	J20.8 (14)	J20.9 (14)	J21.9 (3)			
J39.0 (3)	J41.1 (27)	J41.8 (2)	J44.01 (22)	J44.09 (35)	J69.1 (1)	J69.8 (24)	J85.1 (27)
	J85.2 (7)	J85.3 (11)	J98.50 (15)	J99.8 (187)	K04.7 (5)	K11.2 (6)	K11.3 (1)
	K12.1 (22)	K12.3 (35)	K20 (12)	K29.8 (46)			

	K29.9 (3)	K35.31 (3)	K35.8 (2)	K51.5 (2)	K57.22 (10)	K57.32 (13)	K57.93 (2)	K65.0 (282)
		K65.9 (77)	K67.8 (47)	K75.0 (38)	K81.0 (45)	K83.0 (104)	L02.2 (30)	L02.3 (9)
		L02.4 (31)	L02.8 (3)	L03.01 (4)	L03.10 (8)			
	L03.11 (17)	L03.3 (11)	L03.9 (1)	L04.2 (2)	L08.8 (5)	L08.9 (4)	M00.80 (1)	M00.81 (1)
		M00.91 (4)	M00.92 (2)	M00.96 (6)	M00.99 (2)	M02.89 (1)	M46.22 (1)	M46.42 (5)
		M46.46 (18)	M46.47 (2)	M46.49 (2)	M60.00 (6)			
	M60.99 (2)	M70.2 (3)	M86.97 (4)	M90.29 (2)	M90.88 (2)	N13.6 (20)	N15.10 (5)	N15.11 (1)
		N30.0 (78)	N37.0 (18)	N76.4 (2)	N77.1 (20)	O23.5 (1)	O86.0 (1)	T82.6 (9)
		T82.7 (293)	T83.5 (25)	T84.5 (18)	T85.78 (95)			
	T89.02 (35)	U69.40 (12)	Z22.1 (5)	Z22.8 (17)	Z22.9 (7)	Z86.1 (28)	K52.1 (18)	J22 (18)
Inflammation without infection (843)	G04.8 (25)	G08 (3)	G61.0 (18)	H65.4 (10)	H65.9 (5)	I30.0 (1)	I30.8 (5)	I40.8 (8)
		I68.2 (6)	I77.6 (23)	I80.0 (4)	I80.1 (27)	I80.2 (17)	I80.20 (15)	I80.28 (24)
		I80.3 (7)	I80.8 (6)	I80.80 (22)	I80.81 (18)			
	I80.88 (5)	I80.9 (2)	I83.2 (9)	J31.0 (2)	J32.0 (22)	J32.3 (6)	J32.9 (4)	J37.1 (1)
		J40 (34)	J41.0 (7)	J67.8 (1)	K14.0 (4)	K21.0 (111)	K29.0 (33)	K29.1 (36)
		K29.3 (8)	K29.4 (26)	K29.5 (28)	K29.6 (101)			

	K29.7 (19)	K52.0 (3)	K52.8 (49)	K52.9 (51)	K65.8 (95)	K80.00 (12)	K80.10 (13)	K80.30 (5)	
		K80.40 (1)	K81.1 (11)	K85.10 (4)	K85.11 (6)	K85.20 (5)	K85.21 (11)	K85.80 (18)	
		K85.90 (38)	K85.91 (40)	K86.0 (11)	K86.1 (39)				
	L98.2 (1)	M13.80 (1)	M13.89 (1)	M13.99 (2)	N71.1 (1)	R09.1 (7)	R65.2 (5)	R65.3 (21)	
		R65.9 (5)							
Hemato-	C77.0 (9)	C77.1 (14)	C77.2(25)	C77.3 (3)	C77.4 (6)	C77.5 (6)	C77.9 (4)	C81.4 (2)	
logical		C81.7 (1)	C83.3 (24)	C83.7 (2)	C84.1 (1)	C84.4(4)	C84.5 (3)	C85.1 (11)	C85.9
malignancy (292)	(5)	C88.00 (5)	C90.00 (31)	C90.30 (4)					
	C91.00 (12)	C91.01 (1)	C91.10 (14)	C92.00 (49)	C92.10 (10)	C93.00 (3)	C95.00 (5)	C95.10 (1)	C96.4
	(1)	C96.9 (1)	D46.7 (21)	D46.9 (8)	D47.1 (8)	D47.4 (12)	D61.8 (15)	D61.9 (37)	Z85.6
	(1)	Z85.7 (3)							
Solid	C01 (4)	C05.8 (1)	C08.0 (1)	C08.9 (1)	C10.3 (1)	C10.8 (10)	C11.8 (1)	C11.9 (2)	
Malignancy (428)		C15.1 (10)	C15.3 (3)	C15.4 (7)	C15.5 (16)	C15.8 (6)	C15.9 (7)	C16.0 (12)	
		C16.3 (1)	C16.9 (4)	C17.1 (1)	C17.2 (2)				

	C18.2 (3)	C18.7 (10)	C18.9 (3)	C21.0 (1)	C22.0 (17)	C22.9 (2)	C24.0 (11)	C25.0 (16)
		C25.2 (7)	C25.7 (3)	C31.1 (1)	C34.0 (5)	C34.1 (21)	C34.8 (17)	C34.9 (6)
		C37 (2)	C38.4 (5)	C40.2 (2)	C41.2 (3)			
	C43.6 (1)	C43.7 (5)	C46.0 (3)	C48.8 (1)	C49.4 (4)	C49.9 (5)	C50.8 (12)	C50.9 (8)
		C53.1 (2)	C53.8 (7)	C53.9 (7)	C54.1 (5)	C61 (24)	C62.1 (5)	C62.9 (4)
		C64 (21)	C65 (1)	C67.2 (1)	C67.8 (12)			
	C67.9 (5)	C68.9 (2)	C71.1 (1)	C71.3 (1)	C71.9 (2)	C73 (5)	C74.0 (1)	C76.2 (2)
		C78.0 (37)	C78.2 (5)	C78.4 (3)	C78.5 (5)	C78.6 (35)	C78.8 (9)	C79.3 (22)
		C79.4 (5)	C79.7 (7)	C79.83 (2)	C79.88 (10)			
	C80 (7)	C80.0 (10)	C97 (24)	Z85.0 (21)	Z85.2 (6)	Z85.3 (21)	Z85.4 (17)	Z85.5 (18)
		Z85.8 (11)	C56 (17)	C78.7 (61)				
History of transplantation (316)	Z94.88 (23)	K93.21 (6)	K93.24 (4)	L99.22 (3)	T86.01 (14)	T86.02 (16)	T86.04 (7)	T86.06 (3)
		T86.09 (9)	Z94.80 (11)	Z94.81 (31)	T86.82 (4)	T86.10 (80)	T86.11 (37)	T86.12 (18)
		T86.19 (23)	Z94.0 (127)	T86.81 (3)	Z94.2 (19)			
	Z94.3 (3)	T86.40 (39)	T86.41 (22)	T86.49 (18)	Z94.4 (76)	Z94.1 (9)		

CMV, Cytomegalovirus. EBV, Epstein-Barr virus. HIV, Human immunodeficiency virus. HSV, herpes simplex virus. VZV, varicella-zoster virus. Disease groups and corresponding ICD-10 codes are shown with absolute numbers in parentheses. Due to various numbers of ICD-10 codes in each single patient, patients partially overlap between the disease groups.

Supplement Table S2. Data collection of diagnostic markers.

Diagnostic markers	Time range with regard to maximum ferritin (when not assessed at day of maximum ferritin)
Hemoglobin, platelets, white blood cell count, fibrinogen, albumin [minimum]	± 3 days
aPTT, ALT, AP, AST, bilirubin, creatinine, CRP, INR, lactate, LDH, PCT, ɣGT [maximum]	± 3 days
Triglycerides, max. core body temperature [maximum]	± 5 days

aPTT, activated partial thromboplastin time. ALT, alanine aminotransferase. AP, alkaline phosphatase. AST, aspartate aminotransferase. CRP, c reactive protein. ɣGT, gamma glutamyl transferase. INR, international normalized ratio. LDH, lactate dehydrogenase. PCT, procalcitonin.

Supplement Table S3. Univariable linear regression analyses for the influence of each underlying disease or condition on maximum ferritin.

Independent variable	Regression coefficient	95% CI	P value
Pre-existing immunosuppression	1875.0	707.9, 3042.1	0.002
Tuberculosis	577.7	-5424.3, 6579.7	0.850
Hepatitis	-11.2	-2200.1, 2177.7	0.992
VZV	-56.8	-4888.1, 4774.5	0.982
Malaria	513.8	-12865.4, 13893.0	0.940
HSV	121.9	-2518.2, 2762.0	0.928
Influenza	-20.7	-3571.6, 3530.3	0.991
Liver disease	5378.5	4242.8, 6514.1	<0.001
History of stem cell/ organ transplantation	1939.6	335.8, 3543.4	0.018
CMV	1241.1	-1089.9, 3572.0	0.297
HIV	1030.0	-2209.7, 4269.7	0.533
EBV	1937.4	-2322.7, 6197.5	0.373
Renal disease	311.4	-841.7, 1464.5	0.596
(Bacterial/viral/fungal) infection	-497.4	-2067.2, 1072.4	0.534
Inflammation without infection	-123.6	-1245.6, 998.4	0.829
Autoimmune disease	-1149.4	-3189.1, 890.3	0.269
Solid malignancy	-1158.0	-2572.2, 256.3	0.108

Hematological malignancy	3857.1	2202.3, 5511.8	<0.001

Univariable linear regression analysis was performed separately for each underlying disease or condition (dichotomous no/yes) with maximum ferritin as dependent variable. CI, confidence interval. CMV, cytomegalovirus. EBV, Epstein-Barr virus. HIV, human immunodeficiency virus. HSV, herpes simplex virus. VZV, varicella-zoster virus.

Supplement Table S4. Univariable linear regression analyses for the influence of subgroups of liver disease and hematological malignancy on maximum ferritin.

Independent variable	Regression coefficient	95% CI	P value
K76.1	-1652.6	-6562.4, 3257.3	0.509
Q44.6	-1226.8	-10694.6, 8241.0	0.799
Z75.77	6656.8	1179.5, 12134.2	0.017
K70.0	4595.9	-2836.4, 12028.2	0.225
K70.2	-3617.2	-30359.7, 23125.4	0.791
K70.3	-1441.8	-4129.9, 1246.3	0.293
K70.4	-1412.8	-7569.3, 4743.7	0.653
K70.9	-2697.8	-29440.5, 24044.9	0.843
K71.0	3041.7	-2682.0, 8765.5	0.297
K71.1	2512.5	-9456.2, 14481.2	0.681
K71.7	4343.2	-4126.8, 12813.1	0.315
K71.88	5036.0	-8341.8, 18413.9	0.460
K71.9	579.5	-8348.6, 9507.6	0.899
K72.0	8122.8	6748.4, 9497.2	<0.001
K72.1	9605.8	3892.9, 15318.7	<0.001
K72.71	8965.4	4868.2, 13062.7	<0.001
K72.72	4434.7	326.4, 8542.9	0.034
K72.73	5354.9	1748.4, 8961.3	0.004
K72.74	8927.2	5910.1, 11944.4	<0.001
K72.79	3329.3	1054.8, 5603.8	0.004
K72.9	2975.2	-1337.4, 7287.9	0.176
K74.0	-161.9	-10281.5, 9957.7	0.975
K74.3	-2842.0	-13769.8, 8085.8	0.610
K74.4	3300.0	-8668.4, 15268.4	0.589
K74.5	-2738.4	-21651.8, 16175.1	0.777
K74.6	918.4	-1453.4, 3290.3	0.448
K75.8	1325.4	-7602.6, 10253.4	0.771
K76.0	-315.5	-3169.0, 2538.1	0.828
K76.5	20705.4	13010.3, 28400.6	<0.001
K76.7	763.2	-2202.6, 3729.0	0.614
K76.8	2680.1	-471.9, 5832.1	0.096

K76.9	2094.2	-6377.0, 10565.5	0.628
K77.8	8864.8	-56.8, 17786.4	0.051
O14.2	-3129.7	-18575.3, 12315.8	0.691
R17	-172.1	-7908.7, 7564.4	0.965
C77.0	-2936.0	-11863.4, 5991.4	0.519
C77.1	-2707.8	-9872.4, 4456.8	0.459
C77.2	-2310.3	-7683.2, 3062.6	0.399
C77.3	12584.5	-2853.8, 28022.9	0.110
C77.4	-2281.7	-13209.7, 8646.3	0.682
C77.5	9533.1	-1389.1, 20455.2	0.087
C77.9	-1741.2	-15120.3, 11637.9	0.799
C81.4	-3490.0	-22403.2, 15423.3	0.718
C81.7	-3531.1	-30273.7, 23211.4	0.796
C83.3	4394.2	-1086.6, 9874.9	0.116
C83.7	8605.4	-10305.4, 27516.2	0.372
C84.1	-1943.5	-28686.3, 24799.3	0.887
C84.4	-1636.8	-15015.9, 11742.3	0.810
C84.5	21237.6	5813.3, 36661.8	0.007
C85.1	-1269.0	-9347.8, 6809.8	0.758
C85.9	3427.3	-8541.1, 15395.6	0.574
C88.00	-394.5	-12363.6, 11574.5	0.948
C90.00	4076.0	-752.8, 8904.8	0.098
C90.30	-1590.7	-14969.8, 11788.4	0.816
C91.00	8116.5	386.4, 15846.7	0.040
C91.01	775.5	-25967.4, 27518.4	0.955
C91.10	-97.9	-7263.3, 7067.5	0.979
C92.00	8133.1	4289.5, 11976.8	<0.001
C92.10	1221.2	-7250.3, 9692.7	0.777
C93.00	1784.0	-13661.9, 17229.8	0.821
C95.00	6751.3	-5214.9, 18717.5	0.269
C95.10	-3620.1	-30362.7, 23122.4	0.791
C96.4	-3567.1	-30309.7, 23175.4	0.794
C96.9	-1979.5	-28722.3, 24763.3	0.885
D46.7	8832.4	2983.8, 14681.0	0.003
D46.9	-364.0	-9831.9, 9103.9	0.940
D47.1	-922.7	-10390.6, 8545.1	0.848
D47.4	3805.7	-3929.4, 11540.8	0.335
D61.8	1681.0	-5242.5, 8604.5	0.634
D61.9	4443.2	19.0, 8867.3	0.049
Z85.6	-3386.1	-30128.7, 23356.5	0.804
Z85.7	-3360.3	-18805.8, 12085.1	0.670

Univariable linear regression analysis was performed separately for each subgroup (dichotomous no/yes, represented by respective ICD-10 codes) of liver disease and hematological malignancy with maximum ferritin as dependent variable. CI, confidence interval.

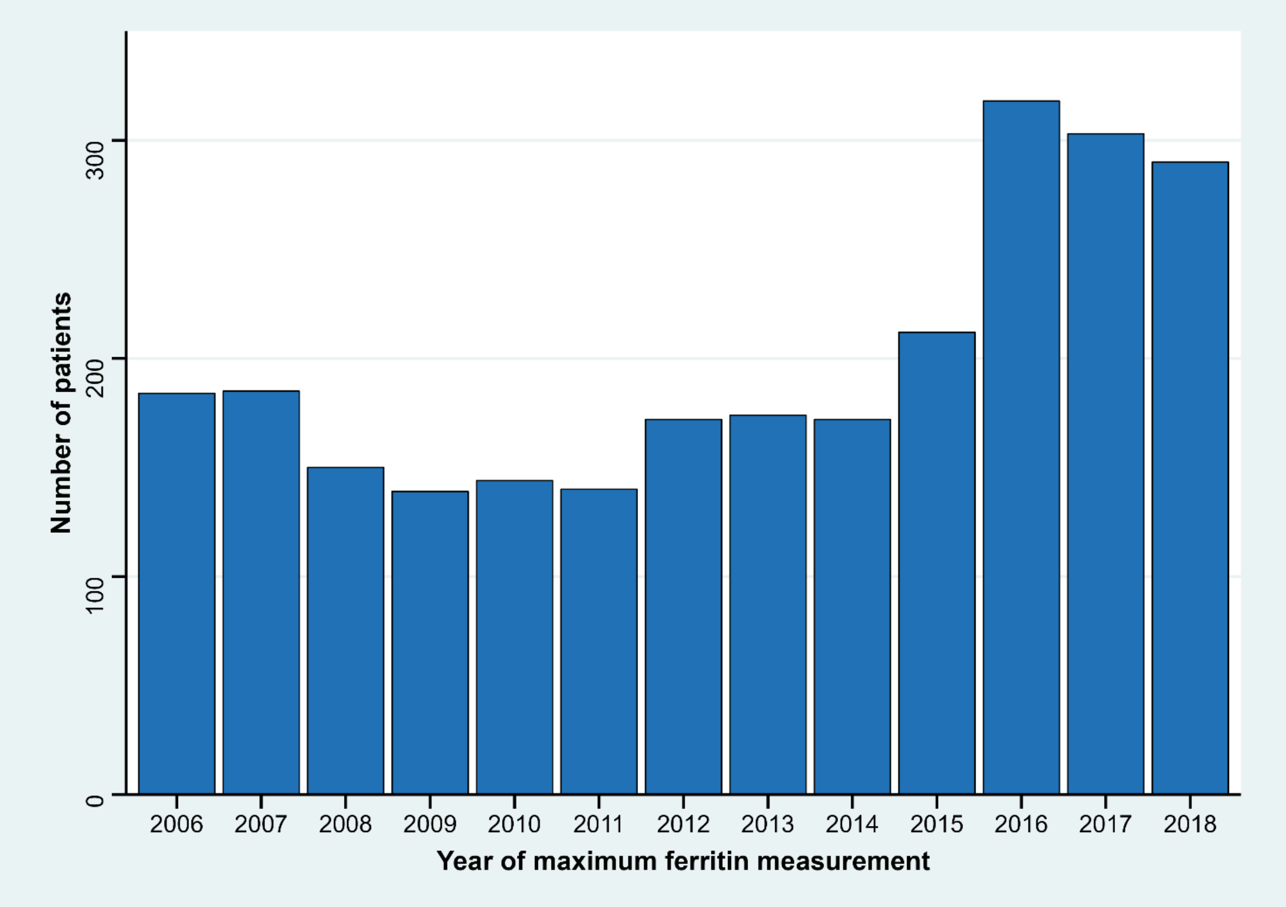

Supplement Figure S1. Distribution of patients with at least one ferritin measurement during ICU course, shown from January 2006 until August 2018.
